# Precision neuropsychology in the area of AI

**DOI:** 10.3389/fpsyg.2025.1537368

**Published:** 2025-05-14

**Authors:** Astri J. Lundervold

**Affiliations:** Department of Biological and Medical Psychology, University of Bergen, Bergen, Norway

**Keywords:** holistic neuropsychology, precision neuropsychology, precision medicine, clinical psychology, artificial intelligence, machine learning

## Abstract

This perspective paper introduces the term “precision neuropsychology” to reflect on an approach that integrates AI-driven assessment tools with traditional neuropsychological frameworks—an integration expected to become crucial in future clinical practice. The paper outlines the technological evolution from basic computerized testing to sophisticated machine learning applications that could enable clinicians to more accurately detect subtypes of neuropsychological conditions. Key opportunities include enhanced pattern recognition in traditional assessments (e.g., digital clock drawing), continuous monitoring of symptom fluctuations (e.g., Attention Deficit Disorder), and personalized assessment and treatment procedures based on individual needs (e.g., learning disorders). The paper also addresses critical implementation challenges: ethical considerations including algorithmic bias and data privacy; balancing quantitative AI analytics with qualitative clinical expertise to avoid reductionism; and developing new competencies for neuropsychologists to effectively integrate AI in their research and clinical work. By providing practical implementation guidelines while preserving holistic patient care, precision neuropsychology shows promise for enhancing both diagnostic accuracy and treatment efficacy in neuropsychological practice.

## Introduction

During the 20th century, the field of clinical neuropsychology has been marked by a fundamental shift in the understanding of brain-behavior function. While earlier approaches were heavily influenced by localizationist theories, advances in neuroscience have revealed that neuropsychological functions emerge from complex interactions between distributed neural networks (Park and Friston, 2013; Brown and Adams, 2023). This shift has been accompanied by a change in assessment methodology, with a move from mainly using standardized test batteries toward process-oriented assessments emphasizing the involvement of underlying cognitive processes and individual differences in test performance (Kaplan, 1988). Prigatano's holistic, multidisciplinary framework for neuropsychological rehabilitation is a good illustration of this process-oriented approach (Prigatano, 1999; Garćıa-Molina and Prigatano, 2022). This multidimensional perspective recognizes that successful rehabilitation requires addressing not just cognitive deficits but also emotional adjustment, awareness of limitations, and social support systems.

The approach has also led to technological innovations that have enriched and challenged the field of psychology during the last decades (Diaz-Orueta et al., 2020; Parsons and Duffield, 2020). These allow for more nuanced understanding of individual differences and better tailoring of interventions to specific patient needs. The emergence of artificial intelligence (AI) and machine learning technologies represents the next frontier in this evolution, offering powerful tools for enhancing assessment precision and treatment personalization. However, the integration of these technologies brings challenges related to complex data interpretation and maintenance of a holistic understanding of patients in clinical decision-making. This may partly explain why there seems to be a greater lag to implement technological innovations in clinical neuropsychology than in related disciplines (Harris et al., 2024). This gap in technological readiness, combined with what has been described as fundamental methodological and theoretical limitations of the field (Péron, 2024), raises important questions about how clinical neuropsychology needs to evolve to maintain its position in modern healthcare.

###  Precision neuropsychology

To contribute to the discussion of the future of clinical neuropsychology, this perspective paper introduces the term *precision neuropsychology*. Through this concept, we aim to inspire reflections on how the holistic tradition of neuropsychology can be preserved and extended by integrating AI-driven tools and applications into a clinical setting.

The term draws inspiration from precision medicine, an approach that seeks to maximize the effectiveness of disease treatment and prevention by accounting for individual variability in genes, environment and lifestyle (Jameson and Longo, 2015). Precision medicine has helped transform theoretical concepts into practical implementable healthcare solutions. Applications range from personalized treatment plans in oncology and cardiology (Mateo et al., 2022; Sun et al., 2023) to prediction of cognitive decline in early stages of neurodegenerative disorders (Veneziani et al., 2024). Recently, there has also been a growing interest in using the term precision psychiatry to emphasize the value of identifying factors that can contribute to personalize treatment for specific patients and diagnostic subgroups (Williams et al., 2024). P4 medicine—defined as Predictive, Preventive, Personalized, and Participatory medicine—represents an expanded framework that builds upon precision medicine principles (Hood, 2017). This framework has advanced substantially in recent years through the integration of AI tools and applications.

Within this emerging precision-oriented landscape, precision neuropsychology may serve as a bridge between traditional neuropsychological and AI-enhanced methodologies. Applying the principles of personalization, prediction, and prevention to neuropsychological practice, may extend our understanding of brain-behavior relationships while preserving the holistic perspective that has long characterized the field.

###  Technological precursors of current and future AI tools

Before examining the integration of AI in neuropsychological practice, it is important to recognize the technological precursors that have created the necessary infrastructure for these advanced applications. These foundational technologies have gradually shifted neuropsychological assessment from purely analog methods to increasingly digital and computational approaches.

The evolution toward digital neuropsychological assessment began with the computerization of traditional paper-and-pencil tests in the 1980s and 1990s. Platforms like Cambridge Neuropsychological Test Automated Battery (CANTAB) (Smith et al., 2013) and the Cogstate (Maruff et al., 2013) standardized administration procedures while offering millisecond precision, automated scoring, and reduced administrator bias (Parsons, 2016). Later developments, such as the NIH Toolbox Cognition Battery, incorporated adaptive testing algorithms, improving measurement efficiency, and reducing floor and ceiling effects (Fox et al., 2022).

Digital assessment expanded available data types and volume. Beyond accuracy and reaction time, newer methods incorporated process data—detailed behavioral patterns captured during task completion (Diaz-Orueta et al., 2020). Ecological momentary assessment (EMA) enabled repeated sampling in real-world environments through smartphone applications, addressing ecological validity limitations of laboratory assessments with moment-to-moment changes in neuropsychological function across different contexts and time scales (see e.g., Harris et al., 2024).

Initial machine learning applications primarily focused on diagnostic classification using supervised learning techniques like support vector machines (SVM) and decision trees to differentiate between clinical populations based on different biomarkers, such as eye-tracking (Bednarik et al., 2013), EEG (Erkan and Kurnaz, 2017), ERP (Mueller et al., 2011), and structural and functional MRI (Orru et al., 2012). SVM and network models are also used in several studies on speech disorders (Brahmi et al., 2024).

Wearable and environmental sensors created new opportunities to assess cognitive and behavioral functioning continuously in naturalistic settings. Accelerometers traced physical activity patterns that correlate with cognitive status and mood fluctuations (Saeb et al., 2015), while actigraphy monitors provided sleep architecture (Ryals et al., 2023). Smart home technologies enabled tracking of activities of daily living (Harris et al., 2024; Hong et al., 2024), and speech analysis identified linguistic markers associated with cognitive impairment (e.g., Olah et al., 2024).

Computerized cognitive remediation programs such as Cogmed, developed by Klingberg (2012), represent important technological precursors that laid groundwork for current AI applications in neuropsychological treatment. These platforms established methodologies for digitally tracking cognitive performance, adapting difficulty levels based on user progress, and generating quantitative outcome metrics.

Traditional statistical methods established conceptual frameworks for understanding complex cognitive data. Latent variable approaches, including factor analysis and structural equation modeling, identifies underlying cognitive constructs (Miyake and Friedman, 2012), while longitudinal analysis techniques like growth curve modeling characterized cognitive trajectories (Wilson et al., 2013). Network analysis conceptualized cognitive systems graphs with interconnected nodes rather than isolated modules (Borsboom and Cramer, 2013), and computational cognitive modeling implemented theoretical processes as executable computer programs that could simulate human performance (Parr et al., 2018).

The convergence of these technological foundations, together with GPU-accelerated computing, created the necessary infrastructure for current AI applications, generating large datasets and establishing frameworks for conceptualizing cognition. Current AI applications thus represent an evolution - the latest development in a decades-long progression toward sophisticated digital approaches to understand brain-behavior relationships.

###  Current AI-tools and applications

Integration of AI and machine learning technologies into clinical neuropsychological practice is currently a critical frontier. Interest in this intersection has grown substantially, as evidenced by recent publications (e.g., Kaur et al., 2025; Tariq, 2025) which are published in a book entitled *Transforming Neuropsychology and Cognitive Psychology With AI and Machine Learning*. A comprehensive review of all current AI studies in neuropsychology is far beyond the scope of the reflections presented in this perspective paper. Only a small fraction of studies will therefore be presented in Table 1, selected to show the large span in studies, from established tools already adopted in clinical settings to promising emerging applications still undergoing final validation. This overview is followed by a more detailed descriptions of three studies to illustrate their methodological rigor, clinical applicability, and potential for immediate implementation.

**Table 1:** AI-tools and applications and their clinical outcomes.

**Tool/application**	**Clinical outcomes**
Digital clock drawing analysis	Improved discrimination between MCI subtypes related to Alzheimer's or Parkinson's disease, with >80% classification accuracy (Wang et al., 2025)
ADHD classification algorithms	Successful identification of ADHD phenotypes with high accuracy; implication for assessment and treatment (Goh et al., 2024)
Continuous monitoring systems	Automatically analyzing streams of behavioral and cognitive data (Chandler et al., 2020)
Linguistic-derived cognitive biomarkers	Early detection of linguistic markers in prodromal Alzheimer's with 88% accuracy (Chou et al., 2024)
AI-based treatment personalization	Potential for optimized intervention selection based on enhanced clinical profiles (review) (Calderone et al., 2024)
VR-based eye-tracking cognitive assessment	Discriminated healthy from cognitive impaired subjects with 88.5% sensitivity, 83% (Xu et al., 2024)
Machine learning for subgroup identification	Predicting transformation from MCI to Alzheimer's disease well beyond chance level (Rye et al., 2022)
Special education AI applications	Enabled targeted interventions for learning disabilities in conditions like dyslexia, ADHD, and social communication disorders (Hopcan et al., 2023)
Risk-calculator for psychosis	The calculator is superior to traditional models to identify at-risk individuals (Krakowski et al., 2024)
Prediction of learning style	Machine learning classifiers with the highest accuracy of 87.5% (Lokare and Jadhav, 2024)

#### Case studies: methodology and results

Several studies have shown the clinical value of a digital version of the Clock Drawing Test. This research provides an excellent example of how traditional neuropsychological tests have been transformed into more powerful diagnostic tools, both between different neurological disorders (Wang et al., 2025), and between patients with a neurological disorder and controls (Binaco et al., 2018). The digital clock drawing test (dCDT) study by Binaco et al. employed multiple machine learning algorithms to classify patients (*n* = 163) with amnestic mild cognitive impairment and Alzheimer's disease based on their performance on a tablet version of the clock drawing task. From this, the researchers captured 350 features including temporal metrics, spatial metrics, and process metrics. Using 5-fold cross-validation to ensure robust performance estimation, they achieved at or above 83% classification accuracy in distinguishing between MCI subgroups and Alzheimer's disease.

The paper by Lundervold et al. (2024) described the power of machine learning in analyzing the construct of psychological distress, defined from five different features (fatigue, anxiety, depression, attention, and memory) in patients with irritable bowel syndrome (IBS). Using Random Forest classification and K-means clustering algorithms, the researchers successfully identified significant patterns in psychological symptoms among IBS patients that traditional statistical approaches might have missed. Their machine learning model correctly predicted IBS diagnosis with 80% accuracy in unseen test data, followed up by an analysis highlighting fatigue and anxiety as the most important predictive features. Furthermore, unsupervised clustering revealed three distinct subgroups of patients with different psychological distress profiles, despite similar IBS symptom severity. This approach uncovered clinically meaningful patient subgroups that could benefit from targeted treatments—one group showed primarily cognitive impairments and anxiety, while another exhibited severe fatigue, sleep disturbances, and depression. These nuanced insights demonstrate how machine learning can detect complex patterns in psychological data that inform more personalized treatment approaches for disorders involving gut-brain interactions. Although not formally implemented in the clinic, it has inspired gastroenterologist to be more aware of individual characteristics when referring patients to treatment.

The article by Goh et al. (2024) demonstrates how machine learning can be powerfully applied to improve ADHD screening and diagnosis. The researchers used random forest regression to identify which ADHD symptoms are most important for predicting future outcomes. From the full set of 18 ADHD symptoms, they identified just eight key symptoms that were most predictive of impairment outcomes five years later. The machine learning algorithm built from these eight core symptoms performed as well as or better than models using all 18 symptoms in predicting global impairment and academic performance. Most impressively, this abbreviated algorithm could predict ADHD diagnosis with 81%–93% accuracy (both concurrently and 5 years later), outperforming current screening tools. Six of the eight key symptoms identified were inattentive symptoms (difficulty sustaining attention, not following through on instructions, poor organization, avoiding mental effort tasks, easily distracted, and forgetfulness), with only two hyperactive/impulsive symptoms (fidgeting and interrupting others). This approach demonstrates machine learning's potential in creating more efficient and accurate clinical screening tools by identifying the most predictive symptoms while eliminating redundancy.

#### Large language models

Large language models (LLM) represent a significant advancement in modern AI, offering new capabilities in clinical documentation, data analysis, and decision support. These artificial intelligence systems, part of the so-called multimodal generative AI technologies, can integrate multiple data streams—combining standard psychometric scores with behavioral observations, neuroimaging findings, and longitudinal monitoring data, and thus serve as supportive tools for clinicians in many ways (Sartori and Orrú, 2023).

**Figure 1:**
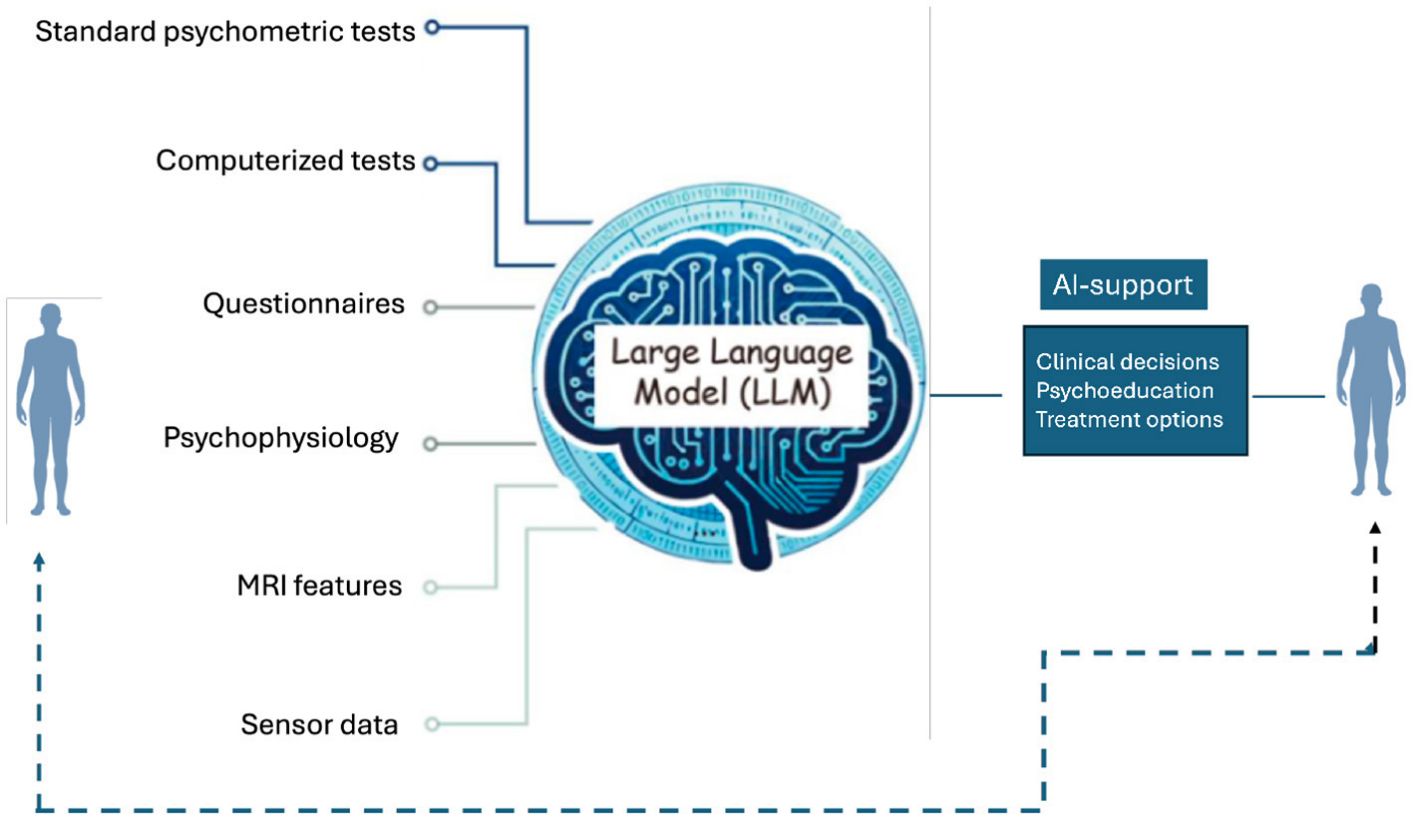
Illustration of how large language models can be used to analyze data from multiple sources and assist in clinical decision making and design of personalized treatment plans. The dashed bidirectional line illustrates that direct human-to-human (patient to the **left**, clinician to the **right**) communication is still important and desirable.

Figure 1 illustrates that direct human-to-human communication remains essential in neuropsychological practice, with AI serving as a supportive tool that enhances rather than diminishes the therapeutic relationship. For a given case, these tools can, for example, be used to integrate various data sources - such as cognitive performance, neuroimaging data, genetic markers, and daily functioning metrics—to provide a comprehensive, updated analysis of treatment response. This integration would enhance treatment effectiveness by coordinating input from multiple specialists while adapting to patient-specific patterns.

Beyond supporting individual clinical analysis, AI tools may also enhance the collaborative aspects of neuropsychological practice. In interdisciplinary teams, where clinical neuropsychologists commonly have a key role (Glen et al., 2019), LLMs can for example be used to streamline the preparation of meeting agendas, automatically distribute relevant documents to participants, and ensure that all team members are well-informed before discussions begin (Lee et al., 2024). AI may also support real-time collaboration by taking notes during meetings and capturing discussions, decisions, actions, and misunderstandings. The immediate distribution of these notes to all team members ensures that everyone remains aligned and that crucial information is not overlooked. This support can facilitate post-meeting actions and assist with follow-ups and information updates across the team. Thus, these technological tools may help neuropsychologists and other health professionals achieve multiple goals simultaneously: enhancing care coordination, using time more efficiently, and making data-driven decisions that can be discussed collaboratively within the group. The result may be a more comprehensive and holistic approach to patient care.

###  New advancements

Although use of AI-inspired digital technology has attracted widespread attention, significant implementation challenges persist, particularly regarding its need for large, diverse data-sets and methods to handle the inherent complexity of neuropsychological data and disorders (Shah et al., 2024). However, innovative AI tools are emerging to address these limitations. One notable example is Retrieval-Augmented Generation (RAG), which extends beyond pre-trained data by accessing external sources such as medical literature, clinical guidelines, and case reports (Yang et al., 2024). This approach offers several advantages: it can include data from groups that are underrepresented in pre-trained data, it provides traceable content for enhanced transparency, and enables more personalized healthcare through integration with medical records and clinical data. Although RAG shows promise, it remains primarily in the research domain and faces its own challenges (Badrulhisham et al., 2024).

Explainable AI is another important advancement in the field of AI-derived analytic tools that are highly relevant for clinical applications (Holzinger et al., 2020). Explainable AI should make it easier for clinicians to understand AI-generated recommendations through transparent visualization of decision paths and contributing factors. This transparency is often seen as essential to integrating AI support with clinical expertise. Several other AI-derived analytic tools are also in the pipeline. To mention one, Feuerriegel et al. (2024) have described a data-driven methods to estimate potential outcomes in response to different treatments. For example, the method can not only predict the risk of transformation from MCI to Alzheimer's disease but also how the risk will change according to available treatments. However, it should be underscored that both these methods need further development before being implemented in clinical practice.

A recent study has also described the possibilities to develop LLMs with more reflexive capabilities (Lewis and Sarkadi, 2024). Such capabilities would allow AI system to monitor and evaluate its own actions and reasoning; consider the potential consequences of its actions; contextualize decisions within a broader ethical, social, and goal oriented frameworks, and learn from experience in a more human-like way.

Finally, Artificial General Intelligence (AGI) should be mentioned as a next frontier in AI development—systems. With human-like general intelligence, it may be capable of understanding, learning, and applying knowledge across diverse domains (Goertzel, 2014). In contrast to current AI systems that are specialized for particular domains, AGI would serve as a universal intellectual amplifier, expanding human cognitive abilities across nearly every discipline. When or if realized, AGI would revolutionize scientific research, personalized education, healthcare, economic planning, environmental management, and creative endeavors.

All these emerging AI capabilities—from AGA to explainable, generative, and reflexive AI—hold significant promise for impacting neuropsychological assessment and treatment approaches in the future. However, they also introduce complex methodological, ethical, and implementation challenges beyond those currently present in clinical practice, requiring thoughtful integration frameworks and ongoing evaluation to realize their full potential.

###  Ethical issues

While precision neuropsychology offers promising advances, success ultimately depends on balancing technological innovation with clinical wisdom. Even sophisticated analytical tools can yield misleading conclusions from incomplete or biased data. AI-derived tools should thus serve as a decision support tool rather than a replacement for clinical judgment. To that end, a neuropsychologist must consider both ethical issues and the need for further education as the field has grown from a niche within computer science to an interdisciplinary endeavor.

A short overview of critical ethical issues is given in Table 2. Not at least, algorithmic bias can amplify existing healthcare disparities through underrepresented datasets. Protecting vulnerable populations, especially children and adults with cognitive impairments, requires protective measures that balance individual safety with equal access to innovative treatments. Informed consent requires particular attention, as healthcare providers must clearly communicate both AI capabilities and limitations while ensuring meaningful patient and family participation in care decisions.

**Table 2:** Ethical issues in AI for clinical neuropsychology.

**Ethical issue**	**Description**
Privacy and data security	Patient neuropsychological data is highly sensitive; AI systems require safeguards against breaches and unauthorized access.
Informed consent	Patients must understand how AI will process their data, what insights might be derived, and any limitations.
Algorithmic bias	AI tools may perform differently across demographic groups if not properly trained on diverse populations.
Transparency and explainability	*Black box* AI decision-making presents challenges for clinicians who need to understand and justify assessments.
Clinical validity	AI tools require rigorous validation against established neuropsychological measures before clinical deployment.
Professional competency decline	Clinicians may become dependent on AI, potentially diminishing their clinical reasoning skills.
Professional boundaries	Questions arise about who bears responsibility when AI contributes to assessment or intervention decisions.
Equitable access	Technology disparities could create or amplify existing healthcare inequalities.
Regulatory challenges	Current frameworks may be inadequate for novel AI applications in neuropsychology.
Human relationship preservation	The therapeutic alliance between clinician and patient could be undermined by technology intermediation.

The risk of precision neuropsychology to oversimplifying human cognition and behavior should also be underscored (Gauld et al., 2024). Current AI tools, while efficient at processing quantitative data, may miss a holistic view on patient needs and experiences, as well as systemic factors, such as family dynamics, cultural context, and life circumstances. Successful AI integration in clinical neuropsychology thus requires educational frameworks that balance knowledge about technological advancements and core clinical competencies. Clinicians must be equipped to critically evaluate AI research, understand methodological and ethical limitations such as algorithmic bias, data privacy, and patient autonomy, and to effectively translate findings into clinical practice (Charow et al., 2021). Since resistance to technological change often stems from valid concerns about maintaining clinical standards, educational programs should demonstrate how AI enhances rather than replaces clinical expertise. The implementation of AI in clinical practice is also expected to face challenges within healthcare systems. In an active clinical practice, easily quantifiable metrics may be preferred over crucial qualitative observations and clinical experiences. Resource constraints and institutional pressure for efficient diagnostics can conflict with a comprehensive assessment. Professional organizations must address these challenges, e.g., by establishing clear AI competency standards, educational programs, and providing implementation support that preserves holistic patient care.

#### The EU AI Act and institutional frameworks

The EU AI Act, formally adopted in March 2024, represents the world's first comprehensive regulatory framework for artificial intelligence (Schuett, 2024). This legislation takes a risk-based approach, categorizing AI systems into four levels of risk: unacceptable (prohibited), high (subject to strict requirements), limited (requiring transparency), and minimal (minimal regulation). For healthcare applications, including neuropsychological tools, many AI systems will likely fall under the high-risk category, requiring robust documentation, human oversight, transparency, and rigorous testing.

While the EU act provides regulatory guidance, institutions must develop their own internal protocols for transparency and data governance (Ning et al., 2024). Even though this legislation establishes a significantly more structured regulatory environment than currently exists in the United States (US), neuropsychology can draw inspiration from comprehensive protocols for AI governance already developed at leading medical institutions (Gupta et al., 2025), for example the Mayo Clinic's framework (Caine et al., 2022; Loufek et al., 2024).

Professional organizations such as the American Psychological Association have also provided guidelines, e.g., related to psychological assessments, but have also raised concerns of unregulated AI technologies (American Psychological Association, 2025). For neuropsychology departments implementing AI systems, existing protocols—including standardized documentation templates, patient consent language, regular audit schedules, staff training requirements, and incident response procedures—can serve as models to be discussed an adapted to current and future ethical and legal issues.

## Summary and conclusion

This perspective paper introduces *precision neuropsychology* as a conceptual framework for reflections on how AI integration with traditional clinical approaches may transform neuropsychological practice. We believe that the rapid evolution of our field, alongside technological innovations in adjacent disciplines, calls for thoughtful discussions about how to integrate AI tools and applications with established neuropsychological principles. This integration may enhance assessment accuracy, enable personalized treatment, facilitate multidisciplinary collaboration, and optimize clinical workflows. Emerging technologies may thus offer opportunities to understand neuropsychological functioning in more authentic contexts than today and support more proactive and personalized models of care (Parsons and Duffield, 2020).

However, implementing precision neuropsychology presents significant challenges. We must address a wide range of ethical considerations, particularly those related to algorithmic biases, data security, and equitable access. The field must resists reductionist tendencies that could undermine holistic patient care while strategically incorporating technological advances to improve outcomes. As AI evolve, clinicians will need to develop new competencies that balance technological literacy with core clinical expertise, including the ability to critically evaluate AI-generated insights (Ringelband and Warneke, 2025).

Looking ahead, we should advocate for a balanced perspective that acknowledges both the promise and limitations of AI in clinical neuropsychology. While the potential for enhancing patient care is substantial, we must maintain critical awareness of the current AI hype cycle. For implementation guidance, neuropsychologists can draw inspiration from institutional frameworks developed in the US, although European practitioners must navigate the more stringent regulations of the EU AI Act. The future of neuropsychology depends on ongoing interdisciplinary dialogue about how to shape our field in an era of continuous innovation, ensuring that these advances enhance rather than replace the irreplaceable human dimensions of neuropsychological practice.

## Data Availability

The original contributions presented in the study are included in the article/supplementary material, further inquiries can be directed to the corresponding author.
